# Quantitative Study on the Breast Density and the Volume of the Mammary Gland According to the Patient’s Age and Breast Quadrant

**DOI:** 10.3390/diagnostics13213343

**Published:** 2023-10-30

**Authors:** Sojin Shim, Jan Unkelbach, Anna Landsmann, Andreas Boss

**Affiliations:** 1 Institute of Diagnostic and Interventional Radiology, University Hospital Zurich, Raemistrasse 100, 8091 Zurich, Switzerland; anna.landsmann@usz.ch (A.L.); andreas.boss@usz.ch (A.B.); 2 Department of Radiation Oncology, University Hospital Zurich, 8091 Zurich, Switzerland; jan.unkelbach@usz.ch

**Keywords:** breast, breast CT, breast density, mammary glands

## Abstract

Objectives: Breast density is considered an independent risk factor for the development of breast cancer. This study aimed to quantitatively assess the percent breast density (PBD) and the mammary glands volume (MGV) according to the patient’s age and breast quadrant. We propose a regression model to estimate PBD and MGV as a function of the patient’s age. Methods: The breast composition in 1027 spiral breast CT (BCT) datasets without soft tissue masses, calcifications, or implants from 517 women (57 ± 8 years) were segmented. The breast tissue volume (BTV), MGV, and PBD of the breasts were measured in the entire breast and each of the four quadrants. The three breast composition features were analyzed in the seven age groups, from 40 to 74 years in 5-year intervals. A logarithmic model was fitted to the BTV, and a multiplicative inverse model to the MGV and PBD as a function of age was established using a least-squares method. Results: The BTV increased from 545 ± 345 to 676 ± 412 cm^3^, and the MGV and PBD decreased from 111 ± 164 to 57 ± 43 cm^3^ and from 21 ± 21 to 11 ± 9%, respectively, from the youngest to the oldest group (*p* < 0.05). The average PBD over all ages were 14 ± 13%. The regression models could predict the BTV, MGV, and PBD based on the patient’s age with residual standard errors of 386 cm^3^, 67 cm^3^, and 13%, respectively. The reduction in MGV and PBD in each quadrant followed the ones in the entire breast. Conclusions: The PBD and MGV computed from BCT examinations provide important information for breast cancer risk assessment in women. The study quantified the breast mammary gland reduction and density decrease over the entire breast. It established mathematical models to estimate the breast composition features—BTV, MGV, and PBD, as a function of the patient’s age.

## 1. Introduction

Breast cancer constitutes more than a quarter of cancer occurrences among women and is the second cancer most frequently leading to a woman’s death [1]. Previous studies widely observed a strong association between breast density, which is the ratio of the amount of fibroglandular tissue in the breast and the amount of fatty tissue, and increased breast cancer risk. While the most important factors for breast cancer risk would be the patient’s age and family history, mammographic breast density is widely considered a strong risk factor for breast cancer that is not specific to the breast side [2,3,4,5,6,7]. Higher density means the glands are located close to each other. This tends to result in more stimulation in glands, which might lead to or be related to breast cancer development. The cancer occurrence rate depends on the anatomical position of the breast according to the breast cancer location database of the Clinical Breast Cancer Project (CBCP) and the Surveillance, Epidemiology, and End Results (SEER) Program of the National Cancer Institute of the United States [8,9]. The upper outer quadrant (UOQ) accommodated tumors from 3.3 to 6.6 times more frequently than other anatomical sites.

Various studies previously assessed the variability of breast density in different radiological breast imaging modalities, the spatial distribution of glandular tissue, and the role of age. For mammography (MG) and ultrasonography (US) examinations, the breast density is visually assessed following the Breast Imaging Reporting and Data System (BI-RADS) classification system of the American College of Radiology (ACR) [4]. In previous studies based on the qualitative BI-RADS density classification in MG, a strong inverse influence of age on breast density was observed [7,10,11]. The conventional breast imaging techniques, however, have limitations in providing quantitative information on breast density or the volume of the mammary glands. The grey level in MG, US, or breast MRI scans represents the relative contrast of either cumulative attenuation, the reflection of the projected beams, or atomic spin density and relaxation time properties but not the real physical tissue density.

In this study, we aimed to assess the breast tissue volume (BTV), mammary glands volume (MGV), and percent breast density (PBD) according to the age of the patient and the anatomical site in the breast. We investigated the variations in the breast composition features with regard to age and breast quadrants. Based on this study’s analysis, we propose regression models to describe these breast composition features according to the patient’s age. Our study assessed the breast composition in volumetric breast images reconstructed by spiral breast computed tomography (BCT) equipped with the latest photon-counting detector. BCT enables true 3D imaging of the breast at an acceptable radiation dose without imposing painful compression on patients’ breasts. The grey level of BCT in the imaging voxel that appears due to the difference in the absorption of glandular and fatty tissue offers a possibility to quantify the amount of glandular tissue and the density and distribution within the breast [12,13,14].

## 2. Materials and Methods

### 2.1. Study Participants

One thousand twenty-seven photon-counting breast CT examinations acquired from women aged between 40 and 74 years from August 2018 to December 2019 were selected for the breast population study. The average age of the women was 57 ± 8 years. We selected patient examinations without breast implants, soft tissue masses, large calcifications (diameter > 1 mm), and resection scars based on the radiological report. This retrospective study was approved by the institutional review board, and written informed consent was obtained from each patient. The participants were grouped into seven five-year intervals based on their age: 40–44, 45–49, 50–54, 55–59, 60–64, 65–69, and 70–74 years old.

### 2.2. Image Data

The breast CT datasets were acquired by a BCT scanner (nu:view, AB-CT—Advanced Breast-CT GmbH, Erlangen, Germany). The patients were examined in the prone position while assuring the central positioning by the radiographers. The detailed image acquisition parameters of the BCT scans and applied dose exposure setup are presented in Appendix A [12,13,14,15,16,17]. The BCT datasets used for this population study were reconstructed with a voxel size of (300 µm)^3^. An exemplary breast CT image screening through the coronal plan can be found in the Multimedia Content.

### 2.3. Image Segmentation and Component Localization

For BCT images, a dedicated segmentation method was developed to classify different breast tissues—the fatty tissues, glandular tissue, and other soft tissues such as the skin and pectoralis major muscle—and automatically localize the quadrants of the breasts [13]. We segmented each breast image into six components by applying the automatic segmentation method [13]: adipose and glandular tissue, skin, pectoralis major muscle, ribs, and skin fold section depicted from the thoracic or abdominal wall. Nipples were segmented as skin. The method was specifically implemented for BCT images using an adaptive region growing algorithm that classifies the glandular tissue by applying the average breast density, which was calculated from the Hounsfield unit (HU) values during the segmentation process, for the voxel-wise probability. In a previous study, this method was validated against human readings and manual segmentations by experienced radiologists as good (4)–excellent (5) in a five-point Likert scale with excellent inter-reader reliability (Cronbach’s alpha test [18], $\rho_{T}$ = 0.83) and a Dice’s similarity coefficient of 0.94 ± 0.04 [13,19].

The four quadrants—upper outer quadrant (UOQ), upper inner quadrant (UIQ), lower outer quadrant (LOQ), and lower inner quadrant (LIQ)—were separated by two perpendicular planes parallel to the row and column of the image intersecting the nipple on the segmented BCT images. The reference nipple position was assigned as the center of mass of the skin in the most posterior slice.

### 2.4. Breast Composition Feature Acquisition

The breast composition features—BTV, MGV, and PBD—were measured on the segmented images. The BTV and MGV were acquired by summing up the volume of the voxels of the adipose and glandular tissues and only the glandular tissue, respectively. The PBD, that is, the percent ratio between MGV and BTV, was calculated from the HU values in the voxels classified as the adipose and glandular tissues [13]. The composition features were acquired for the entire breast and separately for each quadrant.

### 2.5. Breast Composition Feature Analysis

The mean and standard deviation (SD) of the age and the composition features for the entire breast, and each quadrant were acquired for the entire study cohort and each age subgroup. The percentage proportions of the BTV, MGV, and PBD in each quadrant to the entire breast were analyzed in each age group in order to assess the composition variation among the quadrants over the patient’s age.

### 2.6. Statistical Analysis

Differences between mean values were assessed by applying a student’s *t*-test. All reported *p* values are two-sided. *p* < 0.05 was considered to indicate a significant difference. A logarithm model to estimate BTV (1) and a multiplicative inverse model to MGV and PBD (2) were fitted with the least squares method as a function of patients’ age. *a* and *b* are fitting coefficients and constants. *Age*, the predictor, describes the average age of each sub-cohort in years, and *y* denotes the corresponding composition feature as the dependent variable.

(1) y=a×ln(Age)+b

(2) y=a/Age+b

The goodness of the fit for the models was assessed by residual standard error (*RSE*). *RSE* assesses how well a regression model fits a dataset and is acquired by (3), where *SSE* denotes the sum of squares residual error (4) and ${DF}_{SSE}$ degrees of freedom for error (5). ${\hat{y}}_{i}$ is the regressed y of data point *i*, *n* is the number of points in the data sample, and *p* is the number of the independent regressor.

(3) $$RSE=\sqrt{\frac{SSE}{{DF}_{SSE}}}$$

where

(4) $$SSE=\sum {\left(y_{i}-{\hat{y}}_{i}\right)}^{2},$$

(5) $$\;\;\;\;\;\;{DF}_{SSE}=n-p-1,\;i=1,\ldots ,n.$$

## 3. Results

### 3.1. Segmentation and Localization Results

**Figure 1:**
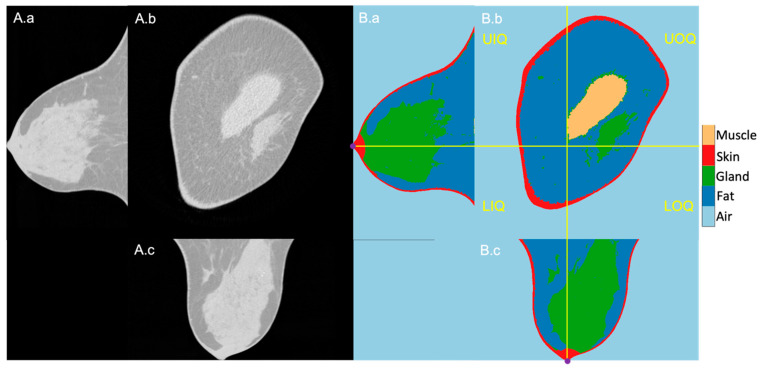
Exemplary BCT original (**A**) and segmentation (**B**) images in longitudinal (**a**), coronal (**b**), and horizontal (**c**) planes. (**B**): From dark to bright grey level, air, fatty tissue, mammary glands, skin, and pectoralis muscle. The four breast quadrants—UIQ, UIQ, LOQ, LIQ—are divided by the perpendicular planes (yellow lines) crossing the nipple position (purple dots in the longitudinal and horizontal planes).

The 1027 BCT images were successfully segmented, and the quadrants could reliably be located based on the nipples that were present at the most posterior slice of the scans. An exemplary image sequence of the original BCT image and the segmentation result of a right-side breast is presented in Figure 1 in three planes: longitudinal, coronal, and horizontal. The reference nipple position is marked as a purple dot, and the perpendicular planes intersecting at the nipple are marked in yellow lines. The right and left sides are the outer and inner sides of the breast, respectively.

### 3.2. Study Participants and Average Composition Feature

The composition features of the selected 1027 breasts—BTV, MGV, and PBD—were successfully measured on the segmented images. On average, the BTV and MGV were 614 ± 388 and 62 ± 68 cm^3^, leading to an average PBD of 14 ± 13% in all breasts.

### 3.3. Composition Feature Analysis across the Age Groups

**Figure 2:**
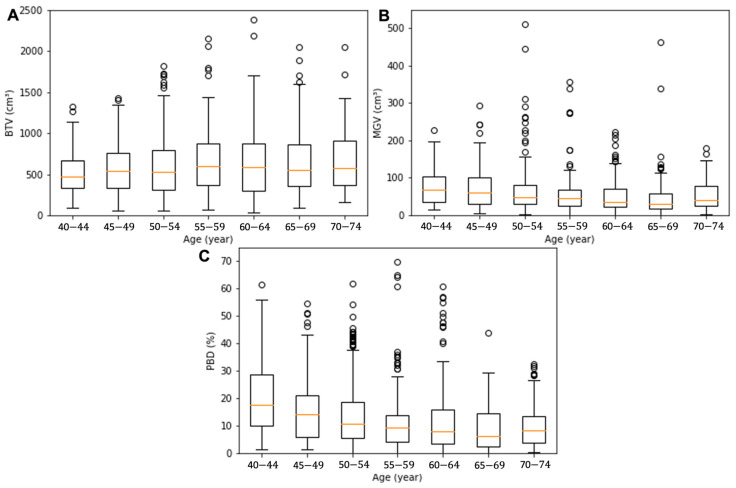
Box and whisker plots for the BTV (**A**), MGV (**B**), and PBD (**C**) of the age sub-cohorts. The orange line in the box denotes the median value, the box the interquartile range (lower quartile to upper quartile), the whiskers the minimum and maximum values of the categories excluding the outliers, and the circles the outliers.

The demographics of the seven groups’ age and breast composition features, which were grouped based on the patient’s age, were analyzed as summarized in Table 1. The average age in each group was 42 ± 1, 47 ± 1, 52 ± 1, 57 ± 1, 62 ± 2, 67 ± 1, and 72 ± 1 year. The BTV presented a gradual increase with age: from the youngest to the oldest, the BTV values were 545 ± 345, 539 ± 314, 589 ± 365, 657 ± 416, 627 ± 401, 663 ± 424, and 676 ± 412 cm^3^. The MGV and PBD presented a decreasing tendency from 111 ± 164, 77 ± 64, 64 ± 59, 56 ± 55, 51 ± 43, 50 ± 57, to 57 ± 43 cm^3^ and from 24 ± 21, 17 ± 15, 14 ± 12, 13 ± 14, 13 ± 13, 10 ± 9, and 11 ± 9%, respectively. The BTV, MGV, and PBD values exhibited a significant difference between the groups with the youngest and oldest patients (*p* = 0.00–0.01). Box and whiskers plots for BTV, MGV, and PBD of the age sub-cohorts are presented in Figure 2. The red line in the box denotes the median value, the box the interquartile range (lower quartile to upper quartile), and the whiskers the minimum and maximum values of the categories excluding the outliers.

**Figure 3:**
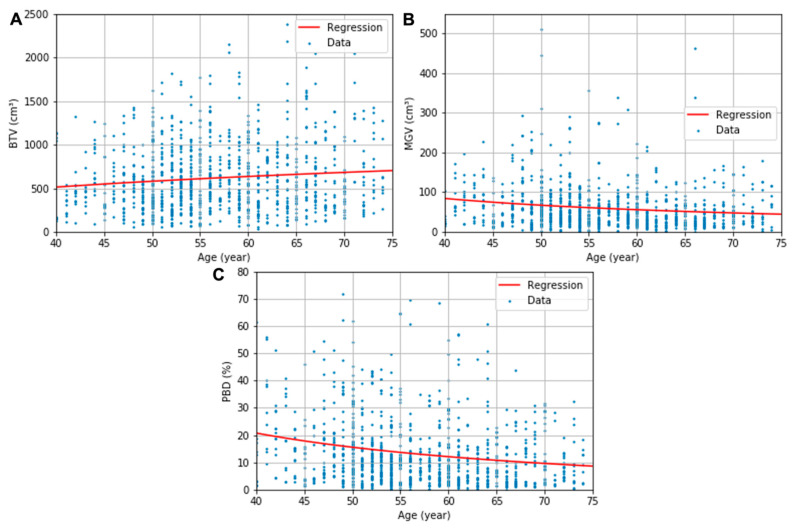
BTV (**A**), MGV (**B**), and PBD (**C**) plots and the corresponding logarithmic or multiplicative inverse model fits as a function of age.

The obtained descriptive estimation models of the BTV, MGV, and PBD as a function of the patient’s age are presented in (6)–(8), respectively. The uncertainty in the regression coefficients and constants were 86 and 345 for (6), 822 and 15 for (7), and 160 and 2.9 for (8), respectively. All regression parameters’ *p* values were below 0.05 except the constants of the regressions for BTV (*p* = 0.11) and MGV (*p* = 0.09). The logarithmic and multiplicative inverse models with the optimized coefficients are fitted in the red line in Figure 3. The regression models’ RSEs were 386 cm^3^, 67 cm^3^, and 13%, respectively, for the *BTV*, *MGV*, and *PBD* plots.

(6) $$BTV\;({cm}^{3})=290\times ln(Age\;(year))-554$$

(7) $$MGV\;({cm}^{3})=4885/Age(year)-26$$

(8) PBD (%)=1237/Age(year)−8.4

### 3.4. Feature Analysis in Each Quadrant

The demographics of the quadrant composition features across all ages are presented in Table 2. The quadrant BTVs in UOQ, UIQ, LOQ, and LIQ were 208 ± 135, 164 ± 107, 126 ± 17, and 117 ± 12 cm^3^, and the MGV were 21 ± 23, 13 ± 15, 17 ± 24, and 12 ± 17 cm^3^. The PBD in UOQ, UIQ, LOQ, and LIQ was 13 ± 13, 11 ± 13, 19 ± 17, and 13 ± 14%. The largest quadrant shares of BTV and MGV were observed in UOQ, which were 34 ± 9 and 34 ± 14% of the breast, and the smallest in LIQ, 19 ± 7 and 18 ± 10%. This led to a similar PBD in UOQ and LIQ quadrants, 13 ± 13 and 13 ± 14%, respectively, which is similar to the entire breast’s PBD. LOQ, on average, presented the highest PBD, 19 ± 17%, with the second biggest MGV quadrant share, 26 ± 13%, and the second smallest BTV share, 19 ± 7%. On the other hand, UIQ is composed of the second largest quadrant share of the BTV, 28 ± 8%, and the second smallest share of the MGV, 21 ± 11%, resulting in the lowest PBD, 11 ± 13%.

### 3.5. Feature Analysis in Each Quadrant across the Age Groups

**Figure 4:**
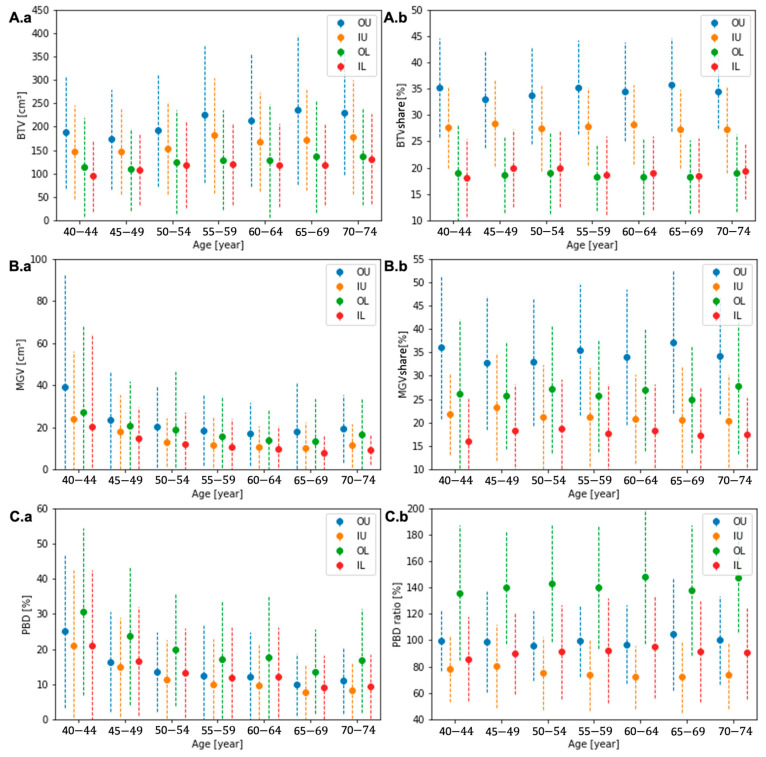
The quadrant BTV (**A.a**), MGV (**B.a**), and PBD (**C.a**) and the quadrant shares of BTV (**A.b**) and MGV (**B.b**) and the ratio of quadrant PBD (**C.b**) to the entire breast for each age group.

Across all groups, the quadrant composition features presented a similar tendency as the entire breast. The average BTC increased, and MGV and PBD decreased in every quadrant with age, as presented in Figure 4A.a, Figure 4B.a, and Figure 4C.a, respectively. The quadrant shares of all three features to the entire breast were relatively stable across all ages, as presented in Figure 4A.b, Figure 4B.b, and Figure 4C.b without a significant variation. Appendix A, Table A2, Table A3 and Table A4, present values of the quadrant composition features and their relative quadrant share compared to the entire breast.

## 4. Discussion

We successfully quantitatively analyzed the breast composition from the 3-D segmentation of BCT images. In this analysis, the breast composition features—BTV, MGV, and PBD—were quantified, and their variation according to the patient’s age and breast quadrants were assessed. The BTV increases, and MGV and PBD decrease with age, exhibiting a significant difference between the youngest and oldest patients’ groups (*p* < 0.05). In the analysis in each quadrant, the largest shares of BTV and MGV were observed in the UOQ, about 34% of the breast, and the smallest shares in the LIQ, 18–19%. The LOQ, on average, exhibited the highest PBD, 1.4 times the mean value for the entire breast, whereas the UIQ only showed three-quarters of the mean value of the entire breast. The BTV increased, and the MGV and PBD decreased homogeneously across quadrants with the patient’s age, which is the same tendency in the entire breast.

The increase in BTV was able to be modeled in a logarithmic function, and the decreases in MGV and PBD in multiplicative inverse functions. Statistical significance in the regressions’ coefficient indicated the general tendency of the breast’s composition features according to the patient’s age (*p* < 0.05), although a significant value for the regression constant of BTV and GTV was not found in this study. When a further study is conducted with larger datasets, leading to a smaller standard deviation in the datasets, a more significant constant for the two models may be acquired in the future. The proposed estimation model’s RSEs—386 cm^3^, 67 cm^3^, and 13% for BTV, MGV, and PBD, respectively—are in the same range of the SDs of composition features in each age group—314–412 cm^3^, 43–164 cm^3^, 9–21%. Although the data were cloudy, which is shown in a relatively large standard deviation of the features in each age group, the mean values contract to the regression curves (see Figure 3). This indicates that the regression curves might show the tendency of the composition in the human body according to age. The strong inverse influence of age on breast density previously observed in the MG studies based on the BI-RADS classification [7,10,11] was systematically proved in our investigation.

We observed a decrease in breast density and amount of mammary gland tissue with patients’ age when the breast cancer risk increases with age. Solely from this observation, the relationship between breast cancer risk and breast density was not confirmed. Breast cancer incidence is a multi-factorial process overlaying different risks. Therefore, in order to study the relationship between cancer risk and a risk factor, the other factors may need to be controlled. For example, the influence of breast density on breast cancer incidence shall be studied in the same age cohort. This needs to be further studied with additional experiments in the future when more patient data is acquired.

Our study demonstrates that the “real” breast density obtained from high-resolution 3D datasets is much lower than the common assumption for mammographic density, 50%, that is applied for the radiation dose analysis and regulation [20,21,22] for mammographic imaging. In our measurements, the PBD in the patient cohort most relevant for mammography imaging between 40- and 75-year-old females was assessed to be 14 ± 13%. The PBDs of all patient sub-cohorts ranged between 11% to 21% from the oldest to the youngest cohort, respectively, which is substantially lower compared to the 50% assumption even for the youngest cohort. The overestimation in mammographic density can possibly lead to a considerable error in radiation dose estimation for MG, where the density assumption plays a critical role. The mean glandular dose (MGD) for MG is commonly assessed by applying the MGD coefficient (DgN) corresponding to 50% breast density following the Dance method [20,23,24], which decreases with an increase in glandularity [25]. An accurate MGD estimation is essential for the assessment of the risk of cancer induction possibly caused by ionizing radiation exposure. Considering the significantly lower breast density of the majority of the patients compared to the assumption in the Dance method, the MGD values of MG might have been underestimated in studies assessing the radiation dose of mammography.

Tumor occurrence is highest in the UOQ (51.5–55.4%), followed by the UIQ (15.6–16.8%), LOQ (10.7–14.2%), LIQ (8.1–8.4%), or center (8.4–10.6%) based on the database in the CBCP and the SEER Program of the United States [8,9]. In these retrospective studies, patients with multicentric disease or breast cancers spanning multiple quadrants were excluded. The assessed quadrant MGV distribution in our study was in line with the previously assessed tumor occurrence rates in the four breast quadrants, as both were the highest in UOQ and the lowest in LOQ. However, a statistical correlation between the MGV distribution and cancer occurrence in the different quadrants was not observed.

Previously, Chen [26] and Fwu [27] estimated the quadrant breast composition using breast MRI images. The BTV, MGV, and PBD of the patient’s breasts were assessed on the segmented breast MRI images by applying a Fuzzy C-means clustering or K-mean clustering algorithm coupled with nonparametric normalization [28]. Chen analyzed 84 cases (47 Asian and 37 Caucasian women) with pathologically confirmed breast cancer, and Fwu did 58 cases of Asian women without a pathological lesion. The analyzed quadrant composition features in the studies were substantially different, which might partially be attributed to the different ethnicities of the cohort. In the studies using MRI images, for example, the largest MGV share and PBD ratio were assessed in the UOQ for the cohort of Caucasian women, whereas they were assessed in the LOQ for the cohorts of Asian women regardless of the presence of pathological lesions. Our study cohort included 517 women without having a pathological lesion and mostly Caucasian and exhibited the largest MGV share in the UOQ and the highest PBD ratio in the LOQ. However, quantification of the quadrant breast composition using the segmented MRI images might have limited accuracy for the analysis. Their composition analysis solely relies on the segmented two-class binary map comprising voxels in 0.7–2.0 mm width, assuming the image has only two discrete true gray levels and ignoring the continuous gray level values due to the partial volume effect in voxels. Furthermore, the statistical segmentation method based on the gray levels representing the relative contrast, which may be distorted by additional signal processing to correct the bias field and intensity nonuniformity, imposes uncertainties in the segmentation result. The estimated breast density based on the image segmentation might substantially vary depending on the algorithm applied, as demonstrated in [29], which assessed the variation of the estimated density up to 10%.

This study has limitations that could potentially be considered as uncertainties in the analysis. Firstly, erroneous segmentation of skin, pectoralis muscle, or skin fold section could cause a bias in the breast composition analysis. In order to minimize possible uncertainty due to the segmentation error, the segmentation method was delicately developed for an accurate segmentation of BCT images that was previously validated against the references by radiologists [13]. Second, the positioning of the patient by the radiographer is crucial to the measured quadrant composition features. Positioning, therefore, could potentially have been a cause of error. However, the segmented images were previously screened to ensure the segmentation quality and the correct nipple position. Third, the patients’ ages were retrieved in natural numbers, not in continuous numbers, as seen in Figure 2, where datasets are plotted in lines instead of distributed in the cloud. This might have imposed an uncertainty on regression model fitting.

The novelty of this study compared to previous mammographic density assessments originates from the quantitative analysis of the breast density and amount of mammary glandular tissue from high-resolution 3D datasets in a large cohort of patients. This study provides further evidence for the previously observed breast density decrease with patients’ age from studies assessing the mammographic density based on the BI-RADS classification [7,10,11]. The study also presents unique anatomical information about how the breast composition is distributed in each quadrant and quantifies the average distribution. The quantitative study applying the diagnostic imager with the latest detector technology even discovered that the real breast density is much less than the present assumption. We are able to further propose a mathematical model to estimate the development of BTV, MGV, and PBD according to age. This estimated breast composition features as a function of the patient’s age provide important data in clinical or scientific assessments of breast imaging, in which breast density influences the diagnostic accuracy or radiation dose exposure. Our data acquired in this study may be further used in models estimating the individual breast cancer risk as breast density is an important independent risk factor for the development of breast cancer [2,3,4,5,6,7].

## 5. Conclusions

This study was able to quantitatively evaluate the amount of glandular tissue and breast density based on the patient’s age from the largest patient cohort ever studied for an epidemiological study using 3D breast images. We demonstrated a statistically significant reduction in the amount of glandular tissue and breast density with the patient’s age and systematically modeled the reduction that allows estimation of the breast density according to the patient’s age. Our study further demonstrates that breast density in women is substantially lower than the commonly accepted present mammographic density assumption. We could quantitatively describe the heterogeneous gland distribution in the breasts with the largest share in UOQ and demonstrate that glands homogeneously reduce across quadrants with the patient’s age. However, in order to quantitatively relate the breast cancer risk and breast density, further controlled studies are required.

## Figures and Tables

**Table 1:** Breast composition features according to age.

		Age Range						
		40–44	45–49	50–54	55–59	60–64	65–69	70–74
Counts		62	120	292	190	176	123	70
Age/1	Mean	42	47	52	57	62	67	72
	SD	1	1	1	1	2	1	1
BTV/cm^3^	Mean	545	539	589	657	627	663	676
	SD	345	314	365	416	401	424	412
MGV/cm^3^	Mean	111	77	64	56	51	50	57
	SD	164	64	59	55	43	57	43
PBD/%	Mean	24	17	14	13	13	10	11
	SD	21	15	12	14	13	9	9

**Table 2:** Quadrant composition features and their shares of the entire breast.

Value		Entire	UOQ	UIQ	LOQ	LIQ
BTV/cm^3^	Mean	614	208	164	126	117
	SD	388	135	107	17	12
Quadrant BTV share/%	Mean	-	34	28	19	19
	SD	-	9	8	7	7
MGV/cm^3^	Mean	62	21	13	17	12
	SD	68	23	15	24	17
Quadrant MGV share/%	Mean	-	34	21	26	18
	SD	-	14	11	13	10
PBD/%	Mean	14	13	11	19	13
	SD	13	13	13	17	14
Quadrant PBD ratio/%	Mean	-	99	75	142	92
	SD	-	31	27	47	37

## Data Availability

The data presented in this study are available on request from the corresponding author. The data are not publicly available due to patient confidentiality.

## Appendix A

**Table A1:** The acquisition parameters of the BCT scans.

Parameters	Value
Focus-Isocenter distance	500 mm
Detector-Isocenter distance	150 mm
Detector pixel size	(100 μm)^2^
Number of projections per 360°	2000
Rotation time	2 s
Focal spot size	0.3 (according to IEC 60336)
X-ray energy	60 kVp
Filtration	3 mm Al
Tube current	32 mA
Total collimation	31.53 mm
Pitch	1.05
Trajectory	Spiral, but circular for the first rotation
Total acquisition time	7 to 12 s

**Table A2:** Quadrant BTV and their share in the entire breast according to age.

Quadrant	Value	Age Range						
		40–44	45–49	50–54	55–59	60–64	65–69	70–74
UOQ/cm^3^	Mean	188	174	193	226	214	236	230
	SD	120	107	119	146	142	159	134
UOQ share/%	Mean	35	33	34	35	34	36	34
	SD	9	9	9	9	9	9	7
UIQ/cm^3^	Mean	146	147	154	181	167	171	178
	SD	101	91	97	123	106	108	122
UIQ share/%	Mean	28	28	27	28	28	27	27
	SD	8	8	8	7	8	7	8
LOQ/cm^3^	Mean	114	109	125	129	128	136	137
	SD	106	88	111	107	121	120	103
LOQ share/%	Mean	19	19	19	18	18	18	19
	SD	9	7	8	6	7	7	7
LIQ/cm^3^	Mean	96	109	118	120	118	119	131
	SD	77	76	92	87	90	86	96
LIQ share/%	Mean	18	20	20	19	19	19	19
	SD	7	7	7	7	7	7	5

**Table A3:** Quadrant MGV and their share in the entire breast according to age.

Quadrant	Value	Age Range						
		40–44	45–49	50–54	55–59	60–64	65–69	70–74
UOQ/cm^3^	Mean	39	23	20	19	17	18	19
	SD	53	23	19	17	15	23	16
UOQ share/%	Mean	36	33	33	35	34	37	34
	SD	15	14	14	14	14	15	12
UIQ/cm^3^	Mean	24	18	13	12	10	10	11
	SD	32	18	11	13	10	12	10
UIQ share/%	Mean	22	23	21	21	21	21	20
	SD	9	11	11	11	10	11	10
LOQ/cm^3^	Mean	27	21	19	15	14	13	17
	SD	42	21	28	19	15	20	17
LOQ share/%	Mean	26	26	27	26	27	25	28
	SD	16	11	14	12	13	11	15
LIQ/cm^3^	Mean	20	15	12	10	10	8	9
	SD	44	15	15	14	11	9	7
LIQ share/%	Mean	16	18	19	18	18	17	17
	SD	9	10	11	10	10	10	8

**Table A4:** The quadrant PBD and their ratio to the entire breast according to age.

Quadrant	Value	Age Range						
		40–44	45–49	50–54	55–59	60–64	65–69	70–74
UOQ/%	Mean	25	16	13	12	12	10	11
	SD	22	14	11	14	13	9	10
UOQ ratio/%	Mean	100	99	96	99	97	104	100
	SD	23	38	27	27	30	42	33
UIQ/%	Mean	21	15	11	10	10	8	8
	SD	22	14	11	13	11	8	8
UIQ ratio/%	Mean	78	80	75	74	72	72	74
	SD	25	32	28	27	24	28	25
LOQ/%	Mean	31	24	20	17	18	14	17
	SD	24	20	16	17	17	12	15
LOQ ratio/%	Mean	136	140	143	140	148	138	147
	SD	51	42	44	46	51	49	41
LIQ/%	Mean	21	17	13	12	12	9	9
	SD	21	15	13	14	14	9	9
LIQ ratio/%	Mean	86	90	91	92	95	91	90
	SD	32	31	36	40	39	38	35
